# The Controlled Release and Prevention of Abdominal Adhesion of Tannic Acid and Mitomycin C-Loaded Thermosensitive Gel

**DOI:** 10.3390/polym15040975

**Published:** 2023-02-16

**Authors:** Youping Li, Gaixia Liu, Mengting Wang, Yuling Zhang, Shiwan You, Jing Zhang, Gang Guo, Bo Han, Le Li, Na Zhao

**Affiliations:** 1 Key Laboratory of Xinjiang Phytomedicine Resource and Utilization, Ministry of Education, School of Pharmacy, Shihezi University, Shihezi 832000, China; 2 State Key Laboratory of Biotherapy and Cancer Center, West China Hospital, Sichuan University, Collaborative Innovation Center for Biotherapy, Chengdu 610041, China; 3 College of Chemistry & Pharmacy, Northwest A&F University, Yangling 712100, China

**Keywords:** thermosensitive gel, polymers, extended and controlled release, postoperative abdominal adhesion, tannic acid, mitomycin C

## Abstract

Postoperative abdominal adhesion is one of the most common complications after abdominal surgery. A single drug or physical barrier treatment does not achieve the ideal anti-adhesion effect. We developed a thermosensitive hydrogel (PPH hydrogel) consisting of poloxamer 407 (P407), poloxamer (P188), and hydroxypropyl methylcellulose (HPMC) co-blended. An injectable thermosensitive TA/MMC-PPH hydrogel was obtained by loading tannic acid (TA) with an anti-inflammatory effect and mitomycin C (MMC), which inhibits fibroblast migration or proliferation. The optimal prescriptions of PPH hydrogels with a suitable gelling time (63 s) at 37 °C was 20% (*w*/*v*) P407, 18% (*w*/*v*) P188, and 0.5% (*w*/*v*) HPMC. The scanning electron microscopy (SEM) revealed that the PPH hydrogel had a three-dimensional mesh structure, which was favorable for drug encapsulation. The PPH hydrogel had a suitable gelation temperature of 33 °C, a high gel strength, and complicated viscosity at 37 °C, according to the rheological analysis. In vitro release studies have shown that the PPH hydrogel could delay the release of TA and MMC and conform to the first-order release rate. Anti-adhesion tests performed on rats in vivo revealed that TA/MMC-PPH hydrogel significantly reduced the risk of postoperative adhesion. In conclusion, the TA/MMC-PPH hydrogel prepared in this study showed an excellent performance in both controlled drug release and anti-adhesive effects. It can be used as a protocol to prevent or reduce postoperative abdominal adhesion.

## 1. Introduction

Postoperative abdominal adhesion (PAA) is one of the common complications after abdominal or pelvic surgery [1,2,3]. The incidence of abdominal adhesion after the laparotomy is even as high as about 90% [4,5]. PAA can cause intestinal obstruction, chronic pain, female infertility, and peritonitis, which can significantly impact the patient’s life and can even be life-threatening in severe cases [6,7,8].

The physical barrier is an effective method to prevent or reduce postoperative abdominal adhesion [9,10]. It can isolate the injury site from the adjacent organs or tissues, reduce the inflammatory exudation of local tissues, and protect the wound from friction [11]. According to the structure, it can be divided into an electrospun fiber membrane [12], hydrogel [13], and absorbable biofilm [14]. The electrostatic spun fiber membrane is a nanofiber hydrogel for the drug release that can extend up to two months. However, most polymer electrospun fibers are hydrophobic and lack biological activity [15]. The technology and material systems of hydrogels have evolved significantly so far, leading to many different multifunctional hydrogels in the form of polymer blends, nanocomposites, nanofibers, protein, and DNA-based hydrogels [16]. Thermosensitive hydrogels have been widely investigated for their unique sol-gel phase-transition behavior and their excellent biocompatibility and biodegradability in the prevention of postoperative adhesion [13,17]. Poloxamer 407 (P407) is one of the most commonly used thermosensitive materials for preparing thermosensitive hydrogels [18,19]. It is a nonionic PEO-PPO-PEO triblock copolymer consisting of a central hydrophobic chain of polypropylene (PPO) and two identical lateral hydrophilic chains of polyethylene oxide (PEO) [20]. Its inverse thermogelling property makes it a flowable solution at room temperature (<25 °C), which can be delivered into the abdominal injury site by injection. After entering the body, it does not require any other stimulation and is transformed into a gel state by the body temperature [21]. However, P407 solutions with concentrations higher than 20% have lower phase transition temperatures (<25 °C) and weaker gel strengths, which cannot meet the requirements for intraperitoneal administration [22]. Therefore, poloxamer (P188) is often used to regulate the phase transition temperature of P407 [23]. Hydroxypropyl methylcellulose (HPMC), an excellent gel-forming agent, can increase the gel strength of P407 [19]. However, the pathogenesis of peritoneal adhesion is complex and changeable, and the ideal anti-adhesion effect may not be achieved by using thermosensitive hydrogel as a physical barrier alone.

Inflammatory exudation following an abdominal injury and the migration and proliferation of fibroblasts play essential roles in forming abdominal adhesion. Inflammatory cells activate fibrinogen activator inhibitors (PAIs) and inhibit fibrinogen activators (t-PAs), disrupting the homeostasis of the fibrinolytic system and leading to fibrin deposition and the eventual formation of adhesion [24,25]. The migration and proliferation of fibroblasts can enhance the expression of fibronectin (FN) and type I collagen (Col-I), promote the deposition of extracellular matrix (ECM), and cause more severe adhesion [26]. Anti-inflammatory, fibrinolytic, and anticoagulant drugs have been used to prevent postoperative adhesion [27,28]. Several reports have highlighted the potential of tannic acid (TA) as an antioxidant and anti-inflammatory agent [29,30]. TA is derived from the natural drug Galla Rhois, which contains 10 catechol or gall tannin moieties per molecule and has a natural scavenging effect on free radicals [31]. Mitomycin C (MMC) is an antimetabolite with inhibitory effects on fibroblast proliferation. It inhibits fibroblast collagen synthesis by suppressing the DNA-dependent RNA synthesis and decreasing the fibrous adhesion [32,33]. Nevertheless, it is limited by the easy and rapid removal of the drug from the body or application site and does not achieve the desired effect.

The trials of various anti-adhesion methods have shown that a single approach does not achieve satisfactory results. Some studies had shown that the combination of the drug and the physical barrier has a better anti-adhesion effect. Chih-Hao Chen et al. [34] loaded the anticancer drug adriamycin (DOX) into an injectable, heat-sensitive poly(N-isopropyl acrylamide)-based hydrogel (HACPN) to control the release of DOX, which improved the effect of intraperitoneal chemotherapy, while preventing postoperative adhesion. Chien-Tzung Chen et al. [35] prepared a hyaluronic acid (HA)/ibuprofen (IBU) (HAI) multifunctional nanofiber membrane (NFMS) by electrospinning and then cross-linked with FeCl3 (HAIFB NFMS) and 1,4-butanediol diglycidyl ether (BDDE) to prepare HAIFB NFMS. HAIFB NFMS, as a carrier and a physical barrier of IBU, has more evident advantages in reducing local inflammation and preventing tendon adhesion, as exhibited by Xiaoling Li et al. [36]. Li Xiaolin et al. wrapped natural anti-oxidative, and anti-inflammatory compounds extracted from Turkish galls (GEA), which were encapsulated into an injectable and biodegradable thermosensitive hydrogel (GEA-NP/H). The GEA-NP/H showed a better anti-adhesion effect than the blank hydrogel in the rat peritoneal injury cecal abrasion model. Yu Wang et al. [37] prepared the chitosan-loaded hydrogel (CS/NAP hydrogel). The three-dimensional mesh structure of P407 can distribute the hydrophilic drug in the free solvent outside the micelles, while the hydrophobic drug is encapsulated inside the micelles. As the gel degrades or dissolves in vivo, the drug is slowly released, extending the retention time in vivo [20,38].

Based on the above, in this study, an orthogonal design method was used to optimize a thermosensitive hydrogel (PPH hydrogel) blended by P407, P188, and HPMC to have a phase transition temperature and a suitable phase transition time following the abdominal temperature. Then, a certain mass of TA and MMC was loaded into the PPH hydrogel to obtain the TA/MMC-PPH hydrogel. The reticular structure of the hydrogel can attenuate the release of TA and MMC and give them a longer duration of action at the damaged site. The hydrogel can serve as both a carrier for the slow release of the drugs and a physical barrier to isolate the damaged spot from the surrounding tissues or organs. In this way, we explored the multifaceted prevention of postoperative abdominal adhesions by drug combinations and drug-barrier combinations.

## 2. Materials and Methods

### 2.1. Materials

Poloxamer 407 (P407) was purchased from Solaibao Biological Technology Co., Ltd. (Beijing, China). Poloxamer 188 (P188) and hydroxypropyl methylcellulose (HPMC) were purchased from Macklin Biochemical Co., Ltd. (Shanghai, China). Tannic acid (TA) was bought from Yuan ye Biotechnology Co., Ltd. (Shanghai, China). Mitomycin C (MMC) was obtained from Jinming Biotechnology Co., Ltd. (Beijing, China). All other chemicals and reagents used were of an analytical grade.

Sprague Dawley (SD) rats (200 g to 250 g) were used in this study and obtained from the Experimental Animal Center of Xinjiang Medical University. All the animals were fed separately with enough food and water at a temperature of 20–24 °C and a relative humidity of 45–55%. The rats were deprived of food for 12 h but had free access to water before the experiments. Animal works were reviewed and approved by the Ethics Committee of the First Affiliated Hospital of Shihezi University (approval number, A-2022-168; 25 November 2022). The animal experiments followed the Guide for the Care and Use of Laboratory Animals (Eighth Edition, 2011).

### 2.2. Orthogonal Experimental Design to Optimize the Prescription of PPH Thermosensitive Hydrogel

Based on the composition of the gel prescription and the results of single-factor experiments, the concentrations of P407 (A) (*w*/*v*), P188 (B) (*w*/*v*), and HPMC (C) (*w*/*v*) were selected as the independent variables (Table 1). The phase transition time of the PPH hydrogel at 37 °C was taken as the dependent variable (a phase transition time of 60–80 s was optimal). The prescription optimization was carried out according to the four-factor, three-level orthogonal table L_9_(3^4^) to determine the optimal process and level.

### 2.3. Preparation of TA/MMC-PPH Thermosensitive Hydrogel

The optimal prescription of the gel was obtained according to the orthogonal experimental design. The hydrogel was prepared according to the “cold method” [39]. The prescribed P407 powder was accurately weighed and 5 mL of cold distilled water was added and stirred well on a magnetic stirrer until a clear and transparent P407 solution was obtained. Then, the prescribed amount of P188 and HPMC powder to was added the solution and stirred well to get a clarified solution. The resulting solution was placed in a refrigerator at 4 °C for 48 h to swell fully, and finally, the purified and transparent PPH hydrogel was obtained. To prepare the TA-PPH, MMC-PPH, and TA/MMC-PPH hydrogel, a specific mass of TA and MMC powder was added to the prepared PPH hydrogel, stirred well, and then fully dissolved at 4 °C refrigerator for 48 h.

### 2.4. Characterization of PPH Thermosensitive Hydrogel

#### 2.4.1. Observation of the Sol-Gel Transition

The vial inversion method studied the sol-gel phase transition behavior and phase transition time of PPH thermosensitive hydrogels [40]. In total, 2 mL of PPH thermosensitive hydrogels were added to 5 mL of filling vials, stored at 4 °C for 10 min, and then placed in a preheated 37 °C constant temperature water bath and inverted after every 15 s to check the sol-gel phase transition. When the hydrogel was observed as unable to flow visually within 30 s, it was considered to be in a “gel” state, and there was recorded as a phase transition time. The phase transition time was recorded by estimating the data from three experiments.

#### 2.4.2. Morphology of Hydrogels

The morphology of the lyophilized hydrogels was observed using scanning electron microscopy (Carl Zeiss AG, Zeiss Sigma 300, Oberkochen, Germany). The hydrogel samples were rapidly lyophilized in liquid nitrogen, dried using a freeze dryer, covered with a thin layer of gold, and finally observed under an accelerating voltage of 3.0 kV.

#### 2.4.3. Rheological Determination

The gelation temperature and the state of the gel at 35 °C at different times were determined using a rheometer (Anton Paar, MCR302, Graz, Austria). First, dynamic rheological studies were carried out on a rheometer with a parallel plate geometry of 35 mm diameter. Time-scan oscillation tests of PPH hydrogels were performed at a 1 mm gap. The strain and frequency were set at 1% and 2 Hz. Temperature scanning tests were performed in the temperature range from 5 to 50 °C to evaluate the thermal properties. The heating rate was maintained at 1 °C min^−1^. The storage modulus (G′), loss modulus (G″), and complex viscosity (η) were recorded.

### 2.5. In Vitro Drug Release Study

The in vitro release behavior of TA and MMC from the TA/MMC-PPH hydrogel was studied using the dialysis bag method. Briefly, 5 mL each of 2 mg/mL TA solution 2 mg/mL MMC solution, and 2 mg/mL TA/MMC-PPH mixed hydrogel were prepared, accurately. Then, 3 mL of these three solutions was taken in a dialysis bag with a cut-off molecular weight of 3000 Da. The dialysis bags were placed in a vessel containing 50 mL of phosphate buffer solution (PBS, pH 7.4) and gently shaken at 100 r/min in a constant temperature water bath shaker at 37 °C. At predetermined time intervals, 1 mL of the release medium was removed from it and replaced with 1 mL of the fresh medium. High performance liquid chromatography (HPLC) determined the collected samples for the concentration of released TA and MMC. The measurements were taken at least in triplicate.

The in vitro release data of the TA/MMC-PPH hydrogel was fitted to the in vitro release models commonly used for extended-release and controlled release formulations to investigate their in vitro release mechanisms and release characteristics. The specific models were as follows:

Zero-order release model:     *Y* = *K*_0_ · t (1)

    First-order release model:      *Y* = 1 − e^−*K*_1_t^ (2)

    Higuchi release model:      *Y* = *K_H_*(t)^1/2^  (3)

    Ritger-Peppas model:         *Y* = *K_RP_*t^n^   (4)

where *Y* is the cumulative percentage release of the drug at time t, *K*_0_ is the zero-order release rate constant, *K*_1_ is the primary release rate constant, *K_H_* is Higuchi’s release rate constant, *K_RP_* is the Ritger-Peppas release rate regular, and n is the release parameter (diffusion index).

### 2.6. Establishment and Grouping of Rat Cecum Abrasion-Abdominal Wall Injury Model

The rats were randomly divided into 6 groups (*n* = 6): sham-operated group, model group, PPH group, TA-PPH group, MMC-PH group, and TA/MMC-PPH group. The rats fasted for 12 h before surgery but had free access to water. Anesthesia was intraperitoneally administered using chloral hydrate (10 wt%) at 3 mL/kg. Then, the abdominal rat hair was shaved and disinfected with an iodine solution. After allowing the iodine solution to dry, the surgical cavity towel was laid. A 3-cm incision was made along the midline of the abdomen with a surgical scalpel under aseptic conditions to find and separate the cecum. The cecum was gently abraded through sandpaper until punctate bleeding was appropriated. The lateral wall of the rat was fully exposed, and a wound of approximately 0.5 mm in depth and 0.5 × 1 cm^2^ in size was first made with a surgical blade at a location approximately 0.5 cm from the edge of the incision. After the appendix was placed back into the abdomen, the midline incision was closed layer-by-layer with 3-0 nylon sutures, and the suture was wiped with iodophor to prevent infection. For the sham-operated group, 3.0 mL of saline was dripped into the abdominal cavity after dissection; the control group flushed the injury site with 3.0 mL of saline after mapping. For the PPH, TA-PPH, MMC-PPH, and TA/MMC-PPH groups, 3 mL of PPH, 3mL of TA-PPH (TA: 100 mg/kg), 3 mL of MMC-PPH, and 3 mL of TA/MMC/PPH (TA: 50 mg/kg, MMC: 10 mg/kg) were injected into the injury site after mammography. After surgery, the animals fasted for 12 h.

### 2.7. Grading of Peritoneal Adhesion in Rats

On postoperative day 7, the rats were euthanized and a U-shaped incision opened the abdominal cavity. The status of intra-abdominal adhesion was assessed according to the scoring system of Nair et al. [41] (Table 2) and photographs were taken.

### 2.8. Statistical Analysis

The results are expressed as the mean ± SD. One-way ANOVA, followed by Tukey’s multiple comparison post hoc test, was used to test the group differences. The experiments were repeated numerous times as independent experiments. The data shown in each figure are a complete dataset from one representative, independent investigation. No samples were excluded from the analysis. The statistical significance is indicated as * *p* < 0.05, ** *p* < 0.01, *** *p* < 0.001, and **** *p* < 0.0001. Statistical analyses were performed using Graph Pad Prism v.8.0 (Graph Pad Software).

## 3. Results and Discussion

### 3.1. Optimization of Proportion of PPH Thermosensitive Hydrogel

Thermosensitive hydrogels should have a suitable gelling temperature (35–37 °C) and gelling time (60–80 s) to prevent abdominal adhesion [42]. Therefore, suitable methods are needed to regulate the gelling temperature and gelling time of P407. The orthogonal experimental design is a simple, fast, and effective multi-factor, multi-level practical design method, in which it is possible to obtain comparable results with many comprehensive tests with a minimum number of tests [43,44]. In this study, using the L_9_(3^4^) orthogonal table, the results for the optimization of the PPH hydrogel were listed in Table S1. The gelling time of PPH hydrogels at 37 °C ranged from 61 to 135 s for each group. The results of ANOVA (Table 3) showed that P407 and P188 had a significant effect on the experimental results (*p* < 0.05), and HPMC had no significant impact on the experimental results (*p* > 0.05). Based on the orthogonal experimental design results, the optimal composition for preparing PPH hydrogels was 20% for the concentration of P407, 18% for the attention of P188, and 0.5% for the concentration of HPMC.

### 3.2. Preparation and Characterization of Hydrogels

#### 3.2.1. Observation of the Sol-Gel Transition Properties and Rheological Properties of Internal Structure of Hydrogels

**Figure 1:**
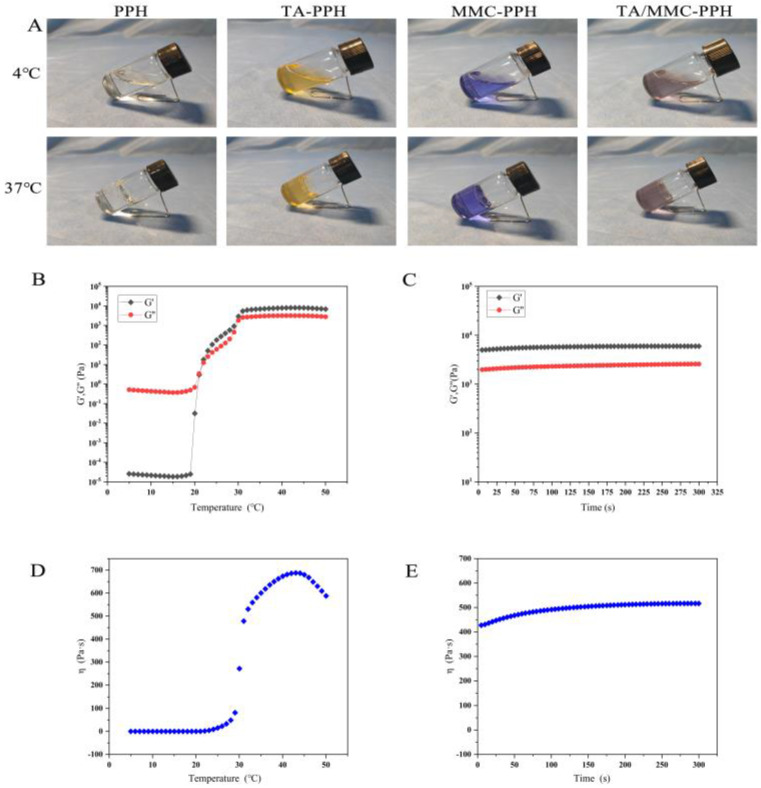
State of each group of hydrogels at 4 °C and 37 °C (**A**) and rheological analysis of the PPH hydrogel. (**B**) Storage modulus (G′) and loss modulus (G″), and (**C**) complex viscosity of the PPH hydrogel as a function of temperature; (**D**) storage modulus (G′) and loss modulus (G″), and (**E**) complex viscosity of the PPH hydrogel as a function of time at 37 °C.

The optimal prescription of the gels was obtained by an orthogonal experimental design. Each group of hydrogels was prepared after the hydrogels were created using the “cold method”, as shown in Figure 1A. We can observe that all four groups of hydrogels are in the flowable solution at 4 °C and in the non-flowable gel at 37 °C. It is possible that with the increase in temperature, the entanglement and stacking between micelles intensify, and the hydrogel undergoes a sol-gel transition [45]. The peritoneal temperature of rats is around 35 °C. Therefore, the injection can deliver the PPH hydrogel to the damaged site in the abdominal cavity. It then quickly transforms into a gel state in the peritoneal cavity, forming a physical barrier to block the damaged area.

**Figure 2:**
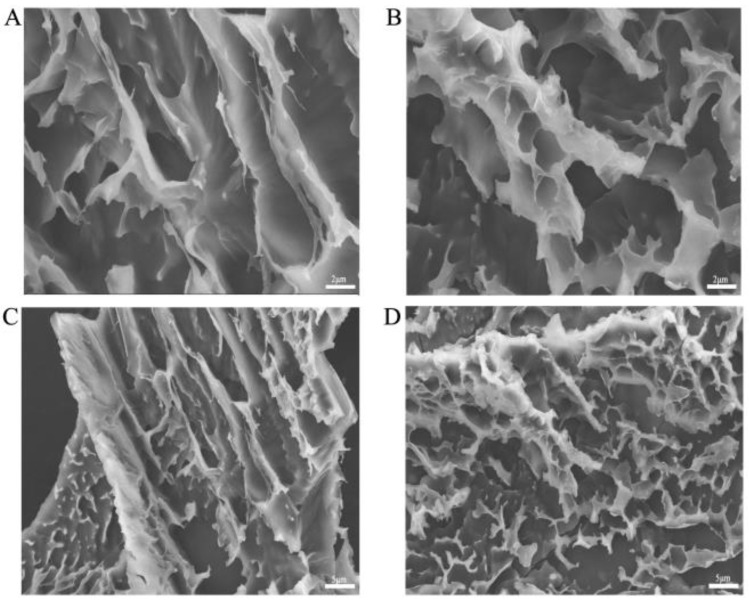
SEM scans of PPH hydrogels (**A**–**D**); scale bar: 2 µm, 5 µm.

The relationship between the magnitude of the elastic modulus (G′) and loss modulus (G″) can reflect the state of the sol-gel and viscosity change of thermosensitive hydrogel [46]. Figure 2 showed the rheological properties of the PPH hydrogel. Firstly, the gelation process of the PPH hydrogel at different temperatures was tested by temperature scan using a rheometer (Figure 1B). At first, the values of both G′ and G″ were small, and G′ was lower than G″, which showed a sol-gel state. In the 20 °C, both G′ and G” climbed in response to the rising temperature, with G″ growing further than G′. When the temperature reached 23 °C, the values of G′ and G″ were equal, which was about 3 Pa, indicating that the micelles started to form inside the PPH hydrogel at that time, and the slow change from sol to gel occurred. Subsequently, the temperature continued to increase, and G″ > G′, but the difference between the two values was insignificant at about 800 Pa. Thus indicated that, although the gelation process had started at that time, the mechanical strength of the gel was not great and did not affect its injectability. When the temperature reached 33 °C, the value of G′ was about 6500 Pa, the value of G″ was about 2700 Pa, and the difference between the two values was close to 4000 Pa. After that, the difference between G′ and G″ remained at 4000 Pa, indicating that at 33 °C, the entanglement and stacking between the hydrogel micelles intensified, and the hydrogel transformed into a high-strength and stable gel state [47]. This temperature can also meet the requirements for intraperitoneal injection drug delivery. Figure 1D shows the variation of η of PPH hydrogel with temperature. We found that the value of η was below 100 Pa·s until 33 °C and tended to be smooth. However, it accelerated sharply when the temperature was at 33 °C, and reached about 300 Pa·s. As the temperature increased, the η value rose and reached 630 Pa·s at 37 °C. At this time, the highly viscous gel could adhere better to the abdominal mucosal tissue [48]. Next, the gelation process and η of the PPH hydrogel at 37 °C were tested by time scan using a rheometer. Figure 1C shows that at 37 °C, G′ (5800 Pa) was always greater than G″ (2400 Pa)m with a difference of about 3400 Pa. The value of η was kept at about 500 Pa·s (Figure 1E). This indicated that the PPH hydrogel presented a high strength and viscosity gel state at the physiological temperature.

#### 3.2.2. Internal Structure of Hydrogels

Scanning electron microscopy observation of PPH hydrogel revealed that the internal structure showed a large number of discontinuous pore structures that were interconnected to form a three-dimensional network structure with a honeycomb-like appearance (Figure 2A–D). This structure facilitates drug encapsulation and delays the release of the drug. It is an excellent vehicle to apply to postoperative abdominal adhesion.

### 3.3. In Vitro Drug Release Studies

**Figure 3:**
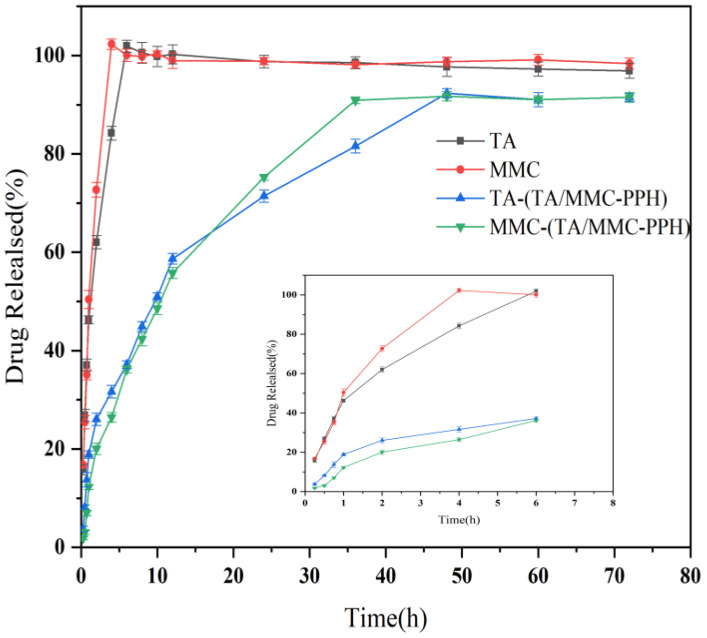
In vitro drug release profile of TA from TA solution and TA/MMC-PPH, and MMC from MMC solution and TA/MMC-PPH. Data are presented as mean ± SD (*n* = 3).

The three-dimensional mesh structure of the hydrogel allows it to encapsulate hydrophilic drugs in the free solvent outside the micelles, while hydrophobic drugs are inside the micelles [46]. Figure 3 shows the release characteristics of TA and MMC in TA/MMC-PPH hydrogel. For the solution of TA and MMC, the release of TA and MMC was almost completed at 6 h and 4 h, respectively. However, the release equilibrium was reached at 48 h and 36 h for TA and MMC in TA/MMC-PPH hydrogel with 92.32% ± 0.97 and 90.89% ± 0.79, respectively, indicating that the PPH hydrogel had an extended and controlled release effect for TA and MMC. From the drug release characteristics in the first 6 h, TA and MMC in TA solution and MMC solution were almost as well wholly released at 6 h and 4 h, respectively. However, the release of TA and MMC in the TA/MMC-PPH hydrogel was 37.16% ± 0.78 and 36.18% ± 0.66, respectively, indicating that this hydrogel can avoid the short-term rapid release of the drug.

The cumulative release percentages of MMC in the TA/MMC-PPH hydrogel were fitted to the zero-order release model, the first-order release model, the Higuchi model, and the Ritger-Peppas model (Table 4). The appropriate model can be judged based on the magnitude of R^2^, which is more significant, indicating a better fit to the release characteristics of the gel [18]. In terms of the release rate, the fit of the first-stage release model (R^2^ = 09929) was better than that of the zero-order release model (R^2^ = 0.7727). It indicated that the PPH hydrogel had better extended and controlled release. Regarding the drug release mechanism, the Higuchi model fit better (R^2^ = 09466), showing that PPH hydrogel had a specific skeletal drug release mechanism and a particular diffusion mechanism for drug release. The diffusion index of the Ritger-Peppas model (*n* = 0.4294) was less than 0.45, indicating that the drug release behavior of the hydrogel was by Fick’s diffusion mechanism (irregular drug transport), and that there was no dissolution effect [49]. The release mechanism of the PPH hydrogel was consistent with the diffusion mechanism.

The occurrence of abdominal adhesions generally occurs 24–36 h after abdominal damage. In vitro release studies have shown that the slow release of TA and MMC from hydrogels is beneficial in preventing the occurrence of adhesions.

### 3.4. In Vivo Anti-Adhesion Study in Rats

**Figure 4:**
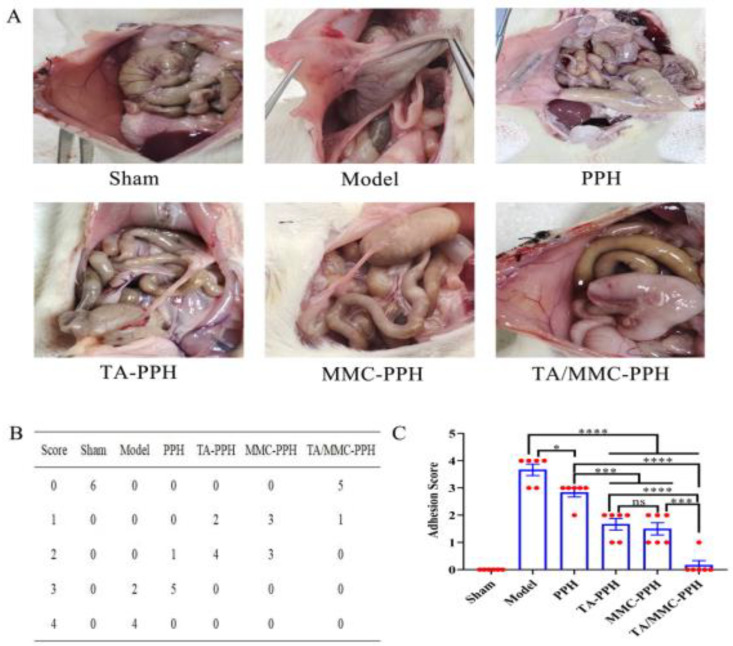
(**A**) Representative pictures of the formation of the abdominal adhesion between the abdominal wall and cecum in the sham-operated group, control group, PPH group, TA-PPH group, MMC-PPH group, and TA/MMC-PPH group at the beginning of the model establishment day 7 post-operation. (**B**,**C**) Adhesion scores of different groups after treatment on day 7 post-operation. (*n* = 6, The red points in the figure represent the number of rats). The data are the mean ± standard deviation (SD) (*n* = 3; * *p* < 0.05, *** *p* < 0.001, **** *p* < 0.0001).

In this study, a rat cecum abrasion-abdominal wall injury model [50] was used to observe the comparative anti-adhesive effects of the sham group, model group, PPH group, TA + PPH group, MMC + PPH group, and TA/MMC + PPH group. The rats were dissected on postoperative day 7, and the degree of adhesion was examined and scored. As shown in Figure 4A, the model group showed severe adhesion on day 7, and the cecum wholly adhered to the abdominal wall. It could not be peeled off with a blunt instrument. The adhesion score was the highest among the groups (Figure 4B,C). Compared to the model group, the PPH group showed weaker adhesion between the abdominal wall and the cecum (Figure 4A), with some of the cecum adhering to the abdominal wall, which could be peeled off with a blunt instrument, and a lower adhesion score than the model group (Figure 4B,C). That may be due to the partial degradation of the PPH hydrogel in the abdominal cavity, which could not completely cover the damaged surface, resulting in adhesion. Observation of the TA-PPH group and MMC-PPH group revealed that the adhesion between the abdominal wall and the cecum was significantly weakened, with only one or two intestinal tracts attached to the surface of the cecum, which could be gently peeled off (Figure 4A). As shown in Figure 4A, the adhesion scores also decreased significantly, mainly with a score of one or two. The TA/MMC-PPH group showed no signs of adhesion, and the traumatized abdominal wall and cecum returned to normal (Figure 4A). Figure 4B,C shows that the TA/MMC-PPH group scored the lowest. There was a large significant difference (*p* < 0.0001) compared to all other groups (except the sham group). That indicates that the PPH hydrogel, combined with TA and MMC, can achieve an excellent anti-adhesive effect. It may be because the PPH hydrogel injected into the damaged surface of the cecum and abdominal wall acts as a physical barrier to block the damaged surface while slowly releasing TA and MMC to inhibit the development of inflammatory response and the migration and proliferation of fibroblasts. The prevention of abdominal adhesion occurs in many ways.

## 4. Conclusions

In this paper, the prescription of PPH thermosensitive hydrogel with a suitable gelling time at 37 °C was optimized by an orthogonal experimental design. The hydrogel had a great sol-gel transition property. It was a flowable solution at 4 °C and a semi-solid gel at 37 °C. At this time, it had high strength and viscosity, which was favorable for attaching to the tissue surface. The simultaneous loading of TA and MMC into this hydrogel resulted in a TA/MMC-PPH hydrogel that was significantly more effective at preventing postoperative abdominal adhesion than their single-use or single combination. Because the three-dimensional mesh structure inside the PPH hydrogel can serve as a drug storage depot, this has a slow-release effect on the release of TA and MMC and prolongs the local drug release time. At the same time, the hydrogel itself can act as a physical barrier to isolate the damaged surface. This creates a multi-pathway of drug combinations, as well as a drug-barrier combination, to prevent the occurrence of a postoperative abdominal adhesion. The TA/MMC-PPH hydrogel prepared in this study was effective at preventing the formation of postoperative abdominal adhesions, and it is hoped that it can also be applied to other forms of adhesions, such as uterine and tendon adhesions.

## Figures and Tables

**Table 1:** Factor and level of orthogonal design (%).

Levels	Factors		
	P407% (A)	P188% (B)	HPMC% (C)
1	18	16	0.3
2	19	17	0.5
3	20	18	0.7

**Table 2:** Adhesion score system for macroscopic evaluation.

Grade	Description of Adhesive Bands
0	Complete absence of adhesion
1	Single band of adhesion, between viscera or from viscera to abdominal wall
2	Two bands, either or from viscera to abdominal wall
3	More than two bands, between viscera or viscera to abdominal wall
4	Viscera directly adherent to abdominal wall, irrespective of number and extent of adhesive bands

**Table 3:** Results of variance analysis of orthogonal design.

Source of Variance	DEVSQ	df	MS	F	*p*
A	2473.56	2.00	1236.78	64.72	0.02
B	881.56	2.00	440.78	23.06	0.04
C	624.89	2.00	312.44	16.35	0.06
D(Errors)	38.22	2.00	19.11		

DEVSQ—the sum of squares of mean deviation, df—degrees of freedom, MS—mean square, F—F text.

**Table 4:** Different release models of hydrogel.

Fitted Model	Fitted Equation	R^2^
Zero-order	*Y* = 21.7496t + 5.5257	0.7727
First-order	*Y* = 92.5121(1 − e^−0.0795t^)	0.9929
Higuchi	*Y* = 11.4527t^1/2^ + 8.0771	0.9466
Ritger-Peppas	*Y* = 16.9150t^0.4262^	0.9484

## Data Availability

Not applicable.

## Supplementary Materials

The following supporting information can be downloaded at: https://www.mdpi.com/article/10.3390/polym15040975/s1. Table S1: Results of Orthogonal design.

## Abbreviation and Nomenclature

Abbreviation and nomenclature	Full Name
P407	Poloxamer 407
P188	Poloxamer 188
HPMC	Hydroxypropyl methylcellulose
PPH	Poloxamer 407, Poloxamer 188 and Hydroxypropyl methylcellulose
MMC	Mitomycin C
TA	Tannic acid
SEM	Scanning electron microscopy
PAA	Postoperative abdominal adhesion
PAI	Plasminogen activator inhibitor
t-PA	Tissue plasminogen active tor
FN	Fibronectin
Col-I	Type I collagen
ECM	Extracellular matrix
HPLC	High performance liquid chromatography
