# Supplementary material for: The Controlled Release and Prevention of Abdominal Adhesion of Tannic Acid and Mitomycin C-Loaded Thermosensitive Gel
Source: Polymers (Basel). 2023 Feb 16;15(4):975. doi: 10.3390/polym15040975 (PMC9966773; doi:10.3390/polym15040975)
Supplement: Supplementary file 1 [file polymers-15-00975-s001.zip › polymers-2137341-SM.pdf]

Table S1. Results of Orthogonal design

Number of tests	A	B	C	Black	Phase transition time（s）
1	1	1	1	1	85
2	1	2	2	2	78
3	1	3	3	3	80
4	2	1	2	3	120
5	2	2	3	1	135
6	2	3	1	2	93
7	3	1	3	2	98
8	3	2	1	3	81
9	3	3	2	1	63
K1	243	303	259	283	
K2	348	294	261	269	
K3	242	236	313	281	
k1	81.00	101.00	87.33	94.33	
k2	116.00	98.00	87.00	89.67	
k3	80.67	78.67	104.33	93.67	
R	35.33	22.33	18.00	4.67	
